# Rehabilitation in Kienböck Disease: A Narrative Review of Current Concepts

**DOI:** 10.3390/jcm15145590

**Published:** 2026-07-16

**Authors:** Dimitar Tenev, Benedikt Hochbein, Georgi Enev, Nikolay Cherkezov, Jakob Adolf, Nikolay Dimitrov

**Affiliations:** 1 Specialized University Hospital for Active Treatment in Orthopaedics “Prof. Boycho Boychev”, Medical University of Sofia, 1614 Sofia, Bulgaria; 2 Centre for Musculoskeletal Surgery, Charité–Universitätsmedizin Berlin, 10117 Berlin, Germany; 3 Department of Consultation Liaison Psychiatry and Psychosomatic Medicine, University Hospital Zurich, 8091 Zurich, Switzerland

**Keywords:** Kienböck disease, lunate osteonecrosis, wrist rehabilitation, musculoskeletal rehabilitation, biomechanics, dart-throwing motion, functional training, sensorimotor control, patient-reported outcome measures, return to work

## Abstract

Kienböck disease, osteonecrosis of the lunate of uncertain aetiology, presents with progressive pain, stiffness, and grip loss in dominant-hand-using adults during their most productive working years, yet its rehabilitation literature has lagged behind its surgical counterpart. This narrative review synthesises current rehabilitation evidence across conservative and post-operative pathways, mapping recommendations to disease stage and to surgical procedure. Following SANRA-aligned methods with selected PRISMA 2020 transparency elements, we appraise immobilisation and orthotic management, dart-throwing-motion-plane mobilisation, sensorimotor retraining, oedema and pain control, psychosocial screening, and procedure-specific protocols that span joint-levelling osteotomy, vascularised bone grafting (including medial femoral trochlea flap), limited intercarpal arthrodeses, proximal row carpectomy, wrist denervation, and salvage arthrodesis or arthroplasty. Patient-reported, performance-based, and occupational outcome instruments are reviewed, with explicit declaration that no Kienböck-validated MCIDs exist. We also set out an integrative reference frame that cross-walks osseous, articular, and tissue-healing axes, offered as clinical orientation rather than as a validated algorithm. The most pressing evidence gaps (Kienböck-specific functional movement screens, sustained return-to-work data, and procedure-specific rehabilitation cohorts) are translated into four registered, powered, COMET-aligned trial proposals.

## 1. Introduction

Osteonecrosis of the lunate was first described by Robert Kienböck in 1910 [1]. The field has remained centred on surgery throughout its development, from Fritz Ståhl’s 1947 monograph on the late results of immobilisation [2], through Lichtman’s radiographic staging [3] and its successive refinements [4,5,6], to the integrated MRI- and arthroscopy-informed classifications now in routine specialist use [7] (Table 1). The patients in question, however, are predominantly dominant-hand-using adults in the third to fifth decades of life, among them manual workers, musicians, and clinicians, in whom modest persistent impairments of grip, pain-free arc, or load-tolerance translate directly into lost work capacity. A nationwide Finnish register analysis reports an annual incidence of fewer than 1 per 100,000 person-years, with approximately half of identified cases proceeding to surgical intervention [8]; comparable register-level data from other countries are scarce, and that figure should be read accordingly.

For the rehabilitation clinician, and for the generalist who may encounter one or two such patients in their career, the surgical literature is paradoxically generous and meagre. Generous, because the technical descriptions of radial shortening osteotomy, capitate shortening, vascularised bone grafting, limited intercarpal arthrodeses, proximal row carpectomy, denervation, and salvage arthroplasty or arthrodesis are extensive [5,6,9]. Meagre, because the postoperative rehabilitation is typically reported in a single sentence of methods, and the conservative-pathway literature, although it includes long-standing observational series showing that a meaningful proportion of unoperated patients reach a symptomatic plateau despite radiographic progression [10,11,12], has not been synthesised in a form that is directly actionable at the bedside. A systematic review by Innes and Strauch concluded that no single surgical procedure has demonstrated superiority [13], a finding consistent with the heterogeneity of pathophysiological models surveyed by Ståhl and colleagues [14]. Rehabilitation guidance accordingly rests largely on expert consensus; the evidence base is at its strongest for early-stage and conservative-pathway decisions, and at its weakest in the late-stage and salvage pathways, where rehabilitation arguably matters most.

Three developments make a focused rehabilitation review timely. First, contemporary staging combines the original radiographic stages [2,3] with perfusion-weighted MRI and arthroscopic cartilage findings [7], offering a biological substrate against which loading and motion progressions can in principle be calibrated, although this integrated approach has not been prospectively validated in rehabilitation cohorts. Second, advances in wrist kinematics and sensorimotor science, in particular the role of the dart-throwing motion plane in everyday activities and the elaboration of dorsal-ligament-mediated proprioceptive reflexes, provide a mechanistic rationale for retraining strategies that depart from conventional flexion-extension drills [15,16]. Third, the wider adoption of validated patient-reported outcome measures [17,18,19] now permits rehabilitation outcomes in this rare disease to be benchmarked across heterogeneous surgical groups in ways that were not previously feasible.

This review pursues two aims. First, to summarise the rehabilitation evidence base for Kienböck disease across the conservative and postoperative pathways, with recommendations mapped to disease stage and to surgical procedure. Second, to synthesise existing biomechanical, sensorimotor, and patient-reported perspectives into a stage-adapted cross-walk intended for clinical orientation and for prospective evaluation, not as a validated algorithm (Section 7). The review is narrative rather than systematic [20]: it does not attempt a meta-analysis, does not undertake a formal risk-of-bias appraisal of every cited primary study, and is explicit at the points where the underlying evidence is sparse. Transparent search and selection methods, intended to mitigate the selection-bias risks inherent to the narrative format, are documented in Section 2.

## 2. Materials and Methods

### 2.1. Reporting Framework and Information Sources

This article is a narrative review; we did not conduct a systematic review. The underlying evidence is predominantly small retrospective cohorts and current-concepts pieces, with few prospective comparisons, and pooled synthesis would be uninformative [21]. We follow SANRA [22] and Ferrari’s narrative-review playbook [23], with selected PRISMA 2020 transparency elements [24], although PRISMA does not formally apply here.

MEDLINE (via PubMed), Scopus, the Cochrane Library and Web of Science were searched from each database’s inception to 17 May 2026; the searches were executed between 18 and 20 May 2026 and were not updated thereafter. Search strings combined controlled vocabulary (MeSH and Emtree) with free-text terms in three concept groups joined with Boolean AND: (i) the disease (Kienböck disease, lunatomalacia, lunate osteonecrosis); (ii) rehabilitation and conservative management (hand therapy, orthoses, range of motion, grip strength, sensorimotor retraining, dart-throwing motion); and (iii) the surgical procedures detailed in Section 5. Reference lists of retrieved reviews, two upper-limb textbook chapters and historical anchor papers were hand-searched.

### 2.2. Inclusion Criteria and Selection Process

Articles in English, German, French, Italian or Spanish were eligible. The Japanese-, Chinese- and Korean-language Kienböck literature was not systematically searched; this is acknowledged as a limitation in Section 8.1. We included primary studies (cohorts, case series and the few comparative trials available), systematic reviews and meta-analyses, current-concepts reviews, and textbook chapters synthesising primary data not otherwise accessible. Case reports were retained only when they illustrated a rehabilitation principle absent from the larger series. Conference abstracts without subsequent full-text publication, unreferenced opinion pieces and animal studies were excluded, except where the latter provided foundational vascular or biomechanical reasoning underpinning current rehabilitation argument. Vocational and return-to-work terms were not embedded in the principal Boolean string; this gap was partially mitigated by hand-searching the rehabilitation-medicine literature on functional capacity evaluation and staged return-to-work pathways.

Titles and abstracts were screened by the first and corresponding author; full texts were independently re-read by a second author before inclusion, with disagreements resolved by discussion. After de-duplication, 1247 unique records were returned; 219 full texts were assessed and 167 were retained for synthesis, supplemented by 16 records identified through hand-searching.

### 2.3. Evidence Appraisal

Each substantive clinical claim in Section 4, Section 5, Section 6 and Section 7 was assigned an evidence level using the 2011 Oxford Centre for Evidence-Based Medicine Levels of Evidence [25] and, where study designs permitted, a GRADE certainty rating [26]; the resulting grades appear in the relevant rows of Table 2 and Table 3. Risk of bias for the non-randomised cohorts on which the procedure-specific sections rely was screened using ROBINS-I [27]. Both appendices were prepared after the narrative drafting was complete, so as not to anchor the synthesis on a particular appraisal verdict. Where a numerical outcome is reproduced from a primary cohort, we flag the absence of dispersion measures when the source paper did not report them.

### 2.4. Reflexivity

Following Sukhera and colleagues [28,29], we acknowledge that evidence selection and weighting in a narrative review are shaped by the authors’ backgrounds. The author group comprises surgeons and clinicians at university clinics (Charité–Universitätsmedizin, Berlin; Universitätsspital Zürich, Zürich and USBALO–“Boycho Boychev”, Sofia) in continental Europe; our routine practice favours early protected motion and dart-throwing-motion-plane sequencing, with consequent attention to those concepts in the synthesis.

## 3. Pathophysiology and Staging, Reframed for the Rehabilitation Reader

Every postoperative loading restriction and every conservative immobilisation decision rests on assumptions about lunate biology, carpal kinematics, and articular surface integrity. The rehabilitating clinician must therefore understand the staging the operating surgeon used and its limitations. This section synthesises pathogenesis, staging, and natural history from a rehabilitation perspective rather than reproducing a surgical primer.

### 3.1. Aetiology: A Multifactorial ‘At-Risk Lunate’

Kienböck’s 1910 monograph described a *traumatische Malazie* of the lunate progressing to compression and collapse [1]. One hundred and fifteen years later the disease retains its original name, but no single sufficient cause has been demonstrated [5,14,30].

Among anatomical factors, negative ulnar variance remains the most debated. Hultén reported an ulnar-minus configuration in roughly three-quarters of affected patients against approximately one-quarter of controls [31], and Palmer and Werner showed that 2.5 mm of ulnar shortening shifts the ulnocarpal load fraction from about 18% to 4% with reciprocal increase across the radiolunate joint [32]. The hypothesis is intuitive but has not held in every cohort: Belgian case-control data found no difference in variance distribution [33], Japanese series have reported positive ulnar variance with clinically indistinguishable presentation [34], and a 2001 meta-analysis returned a non-significant pooled odds ratio of 3.10 for negative versus neutral or positive ulnar variance (95% CI 0.95 to 10.05; *p* = 0.06) [35]. Stahl et al. 2012 [14] systematic review concluded that, in aggregate, the evidence does not satisfy the Bradford-Hill criteria for causation; the Japanese sceptical tradition of the Hultén hypothesis, anchored on Tsuge in the late 1970s and consolidated in Tsuge et al. 1993 [36], has been more emphatic on this point than the Atlantic-rim consensus that the manuscript otherwise inherits [14].

Vascular anatomy is better established as a contributor than as a cause: Gelberman’s injection study of 35 cadaver lunates showed that 7–26% have effectively single-vessel supply [37]. A first population-based signal for familial aggregation from the Utah Population Database is consistent with a multifactorial rather than Mendelian model [38].

For the rehabilitating clinician the message is pragmatic: no programme can claim to address ’the cause’. Programmes address downstream consequences (pain, oedema, lost motion, lost grip, sensorimotor disorganization) and must adapt to a lunate whose biological reserve may be limited.

### 3.2. The Lichtman Radiographic Staging System and Its Modifications

The four-stage radiographic system introduced by Lichtman and colleagues in 1977, originally devised to align outcomes after silicone arthroplasty with preoperative status, has become the standard descriptive language of the field [3]. In its current form: Stage I: normal radiographs with positive MRI or bone-scan signal change; Stage II: lunate sclerosis without collapse; Stage IIIA: lunate collapse without carpal collapse (radioscaphoid angle < 60°); Stage IIIB: lunate collapse with fixed scaphoid rotation (radioscaphoid angle ≥ 60°) and carpal collapse; Stage IIIC: coronal fracture of the lunate, added in the 2010 update [4]; Stage IV: stage III changes with radiocarpal and/or midcarpal arthrosis.

The Goldfarb modification introduced the 60° radioscaphoid-angle threshold separating IIIA from IIIB after the original stage III distinction was shown to be unreliable. With four raters and 39 wrists, Goldfarb and colleagues reported an interobserver κ of 0.63 overall (0.38 for stage IIIA) under the original system, improving to 0.81 (0.75 for stage IIIA) under the angle-based modification [39]. These are point estimates; the original publication does not report 95% confidence intervals, and the four-rater design limits the inferential weight. For the rehabilitating clinician, the substaging matters because carpal load-bearing capacity, proximal-row instability, and scaphoid rotation differ substantively between IIIA and IIIB, and again with the coronal-fracture IIIC.

### 3.3. Functional MRI (Schmitt) and Articular (Bain and Begg) Staging

Two complementary classifications add to the morphological Lichtman scheme. Contrast-enhanced MRI staging assesses lunate viability rather than shape. Schmitt and colleagues’ 1998 German-language paper first correlated MRI signal patterns with the Lichtman stages using gadolinium-enhanced T1, fat-suppressed T2, and bone-marrow-oedema sequences [40]; the same group’s European Radiology paper proposed the three-stage perfusion classification now in common use: A (complete gadolinium enhancement, viable), B (enhancement restricted to a distal reparative zone with non-enhancing proximal pole, partial necrosis), and C (no enhancement, complete necrosis) [41]. This was later consolidated by the Bain group [7]. Because lunate-preserving procedures depend on revascularisable bone, Schmitt A or B is a prerequisite. For rehabilitation, the Schmitt stage signals biological risk: a stage A lunate may tolerate progressive loading after joint-levelling, whereas a partially necrotic stage B warrants more cautious early-phase loading. The relationship between Lichtman IIIB and Schmitt B-C is probabilistic, not categorical [6,7].

Bain and Begg’s consolidation builds on a wrist-arthroscopy methodology developed by Watanabe M. in the 1980s in Japan, which preceded the European systematisation by more than a decade. The articular classification counts the number of non-functional articular surfaces among the proximal pole of the lunate, the distal lunate, the lunate fossa of the radius, and the proximal pole of the capitate, from grade 0 (all four functional) to grade 4 (none functional) [42]; the same group’s 2011 articular-based staging extended this into a three-phase vascular/osseous/chondral model [43]. For the rehabilitating clinician, the grading predicts which articulations remain weight-bearing, which is central to planning loading progressions after scaphocapitate fusion, proximal-row carpectomy, or total wrist procedures.

### 3.4. Integrating the Three Classifications

The 21st-century algorithm of Lichtman, Pientka and Bain integrates the Lichtman morphological, Schmitt perfusion, and Bain articular classifications into a single decision pathway [6]. By addressing simultaneously what the lunate looks like, how alive it is, and how intact its articular surfaces are, the surgeon can match procedure to biology more precisely than any single classification permits.

For rehabilitation, the integrated view yields three working tiers. Early disease with a viable lunate (Lichtman I–II, Schmitt A, Bain 0–1) admits conservative immobilisation, core decompression, joint-levelling, or vascularised grafting; rehabilitation emphasises load reduction and protection of biological reserve. Intermediate disease with a partially viable lunate and preserved cartilage (Lichtman IIIA, Schmitt B, Bain 1–2) directs surgery toward lunate-preserving but unloading strategies, and rehabilitation must protect both construct and lunate. Advanced disease with a non-viable lunate or grossly damaged surfaces (Lichtman IIIB–IV, Schmitt C, Bain 3–4) moves surgery toward lunate-bypassing or salvage procedures; rehabilitation shifts from biological protection to maximising function within altered kinematics. The most recent integrated synthesis [5] refines several decision nodes of the 2017 algorithm [6], most explicitly the placement of revascularisation candidates within Schmitt B and the lunate-preserving versus lunate-bypassing threshold at IIIA–IIIB; the present cross-walk in Table 1 adopts the 2025 placement and flags where it diverges from the 2017 version. The framework is an orientation, not an algorithm, and so surgeon training, occupational demand, patient preference, and ulnar variance may modify decisions, and many patients do not fit neatly into one tier.

**Table 1:** Lichtman, Schmitt and Bain staging systems cross-walked for the rehabilitating clinician.

Radiographic Stage	Radiographic Hallmark	MRI-Perfusion Stage	Articular Stage	Rehabilitation Implication
I	Normal radiograph; MRI bone-marrow oedema or bone-scan signal	A (full enhancement, viable)	Vascular phase; 0 (all four surfaces intact)	Protect biological reserve; load reduction with active mobilisation
II	Lunate sclerosis without collapse	A to B (transition)	Vascular to osseous transition; 0–1	Conservative trial; joint-levelling or VBG candidate
IIIA	Lunate collapse; radioscaphoid angle < 60°; no fixed scaphoid rotation	A or B	Osseous phase; 1–2	Lunate-preserving surgery feasible; rehabilitation protects construct and lunate
IIIB	Lunate collapse; radioscaphoid angle ≥ 60°; fixed scaphoid rotation and carpal collapse	B or C	Osseous to chondral; 2–3	Lunate-bypassing surgery typically required; rehabilitation reorients to altered kinematics
IIIC	Coronal fracture of the lunate (2010 update)	B or C	Chondral phase; 2–3	Descriptive variant of IIIB; salvage-leaning decisions
IV	Stage III changes with radiocarpal and/or midcarpal arthrosis	C (no enhancement)	Chondral/pan-carpal; 3–4	Salvage (PRC, 4CF, TWA-Arthrodesis, TWA-Arthroplasty); rehabilitation focuses on function within altered or absent wrist motion

### 3.5. Natural History: A Caveat Against Surgical Urgency

In untreated cohorts the disease is not uniformly progressive at the clinical level. Kristensen and colleagues, following 49 wrists (23 plaster-immobilised, 26 untreated), reported that 83% (19/23, Wilson 95% CI 64–93%) and 77% (20/26, Wilson 95% CI 58–89%), respectively, were pain-free or experienced pain only with heavy work at follow-up, despite radiographic lunate deformation in all 49 cases and radiocarpal arthrosis in 67% [10]. Beckenbaugh’s cohort reached a comparable conclusion, that symptomatic outcome and radiographic appearance dissociate at long follow-up [11], and the Allan, Joshi and Lichtman review consolidated this into the modern consensus that surgical and non-surgical pathways can converge on acceptable function in selected patients [12]. These data carry methodological limitations (point estimates without confidence intervals, subjective pain assessment, no randomised comparator, pre-PRWE instruments), but they remain the most important counterweight to the surgically urgent narrative that staging diagrams can inadvertently convey: for the apprehensive young patient newly diagnosed at stage II, the radiographic future is bleaker than the symptomatic future in a meaningful proportion of cases.

### 3.6. Wrist Kinematics Relevant to Rehabilitation

Most functional activities of daily living occur along the oblique dart-throwing motion (DTM) plane from radial extension to ulnar flexion rather than along pure flexion-extension or radial-ulnar deviation [44,45]. Crisco and colleagues showed, using markerless bone-registration imaging, that in this plane the proximal carpal row remains relatively stationary while motion concentrates at the midcarpal joint, sparing scapholunate and lunotriquetral relative motion [15]; subsequent MRI and IFSSH-consensus work has refined the operational definition [46,47].

For Kienböck rehabilitation, DTM-plane mobilisation may permit functional motion without imposing the lunate shear of pure anatomical-plane motion; orthoses that block flexion-extension but permit the DTM support task practice without removing lunate protection; and sensorimotor retraining can be sequenced along the DTM as the natural axis of dynamic stabilisation [16]. These ideas have been developed most fully for scapholunate ligament injury, and Kienböck-specific outcome data remain absent. The conceptual case is strong, the empirical case is awaited, and Section 4, Section 5 and Section 7 are explicit about that limitation.

## 4. Rehabilitation in Conservative Management

Conservative management is the entry point for early Kienböck disease, the most realistic option for paediatric and elderly cohorts, and the default when surgical capacity or patient preference precludes intervention. The current-concepts review reaffirms that children, adolescents, and older adults respond well to non-operative treatment [5]. The systematic review of Innes and Strauch found that in mixed adult cohorts conservative care produced the smallest objective gains in grip strength compared with most surgical options, while still yielding subjective pain relief in late-stage groups [13]. The findings are not contradictory; they describe different populations. Kristensen’s long-term cohort, in which a substantial proportion of non-operatively managed patients reached a symptomatic plateau despite radiographic progression, further argues against pure radiographic surveillance as a treatment endpoint [10,14].

### 4.1. Immobilisation: Indications, Materials, and Duration

The Lichtman et al. 1977 [3] series is the historical anchor, but its finding is associational: progressive collapse occurred despite prolonged plaster immobilisation, not because of it [3]. The conventional three-month trial derives from expert-opinion guidance rather than randomised data [9,12], and the systematic review of immobilisation in early-stage disease found limited support for any particular duration [48]. We recommend a pragmatic 6–12 week trial of a forearm-based custom thermoplastic volar wrist orthosis in 0–15° of extension, metacarpophalangeal joints and thumb free, fabricated to permit daily skin checks and gentle digital motion. Plaster remains acceptable where compliance is uncertain. Weaning proceeds over a further 6–12 weeks to night-only or activity-only protection, with re-evaluation at 3 and 6 months. Prolonged static immobilisation impairs proprioceptive performance [16,49]; protected motion in the dart-thrower’s plane from week 3–4 may mitigate this without compromising lunate protection, although Kienböck-specific data are absent and the recommendation is extrapolated [44].

### 4.2. Activity Modification

Activity modification is the conservative pillar with the largest plausible biological effect and the smallest amount of literature. Patients with hand-intensive occupations (carpenters, dentists, musicians, surgeons) and high-load recreation, climbing in particular [50], are advised to reduce loaded wrist extension and gripping in radial deviation, with progression governed by symptoms and serial imaging [51]. Where hand-arm vibration exposure is relevant, EN ISO 5349 action and limit values provide a documentable vocational-counselling framework [52]. Consistent with the sceptical reading developed in Section 3.1, negative ulnar variance is neither necessary nor sufficient for Kienböck disease; the historical association nonetheless warrants attention to ulnar-sided loading patterns [31]. Formal occupational therapy input helps patients distinguish sustainable modification from deconditioning avoidance.

### 4.3. Pharmacological and Adjunctive Measures

Non-steroidal anti-inflammatory drugs are appropriate for symptomatic management subject to standard precautions; they do not appear to alter disease progression. For all other adjunctive modalities the evidence base is materially weaker than narrative reviews have typically implied, and the language below follows the principle that recommendations should accurately reflect best available evidence [53].

Bisphosphonates: No randomised or comparative trial of bisphosphonate therapy specifically in Kienböck disease has been published. Available data are limited to extrapolation from femoral-head osteonecrosis, where anatomy and mechanical loading differ substantially [54]. There is insufficient evidence to recommend bisphosphonate therapy for Kienböck disease.

Pulsed electromagnetic field (PEMF) therapy: No primary clinical study of PEMF specifically in Kienböck disease was identified through the PubMed search. The data are insufficient to recommend PEMF in Kienböck-specific cohorts.

Extracorporeal shock wave therapy (ESWT): Evidence rests on a systematic review of bone-marrow oedema across mixed anatomical sites, in which Kienböck is not analysed as a separate subgroup [55]. No Kienböck-specific prospective trial has been published; the data are insufficient to recommend ESWT outside research protocols.

Hyperbaric oxygen therapy (HBO): Evidence is restricted to two single-case reports of stage III disease [56]. The base is anecdotal (OCEBM Level 5); HBO cannot be recommended outside an investigational context.

### 4.4. Pain and Oedema Control

Low-grade oedema is common and contributes to stiffness. Standard hand-therapy protocols apply, with doses described in the certified hand-therapy literature [57,58]. Coban self-adherent compressive wrapping distal-to-proximal at light tension, changed daily, reduces upper-limb oedema [59]; manual oedema mobilisation, short-stretch retrograde technique and night compression gloves are supported in mixed cohorts [60,61]. Contrast baths (alternating 38–40 °C and 18–20 °C for approximately 3 and 1 min respectively over 20 min) provide modest benefit where no vascular contraindication exists [62]. Cryotherapy 10–15 min after activity is conventional. For pain, graded activity pacing and gentle isometric strengthening within pain-free ranges are appropriate; parameters are extrapolated from the broader distal-radius literature [63,64].

### 4.5. Range-of-Motion and Strengthening Progressions

Motion of unaffected joints (shoulder, elbow, digits) begins on day one. Wrist motion is introduced at 4–6 weeks. Flexion-extension and radial-ulnar deviation impose greater shear on the lunate than dart-thrower’s-plane motion; active and active-assisted dart-thrower’s-plane motion is therefore introduced before conventional anatomic planes [45], with functional cueing analogues (hammering, pouring, dial-turning) [51]. Strengthening begins isometrically in neutral wrist; progression to dynamic loading is governed by a multi-criterion gate rather than a single grip percentage: pain-free at rest and symmetric dart-thrower’s-plane motion and grip ≥ 75% of contralateral and activity VAS ≤ 2/10. Dosing is short and frequent (e.g., four 5-min home sessions daily), reflecting motor-learning rather than gym-physiology principles.

### 4.6. Sensorimotor and Proprioceptive Retraining

Mechanoreceptors in the dorsal wrist ligaments provide afferent signals to the dynamic stabilisers (flexor carpi radialis, flexor carpi ulnaris, extensor carpi ulnaris); immobilisation and pain-related guarding disrupt this system [16,49]. Staged sensorimotor retraining proceeds from joint-position-sense drills through conscious co-contraction and dynamic stabilisation against graded perturbation to reactive neuromuscular tasks. This progression is adapted from wrist-proprioception and scapholunate rehabilitation rather than from Kienböck-specific trials, and is offered as expert-derived guidance. Surface electromyographic biofeedback can guide selective dynamic-stabiliser recruitment, but Kienböck-specific outcome evidence is absent; the modality is adjunctive rather than first-line.

### 4.7. When to Escalate to Surgery

A conservative trial of 3–6 months is conventional expert consensus [9,12]. Triggers for escalation are: failure of pain to improve below a clinically meaningful threshold (≥20 mm reduction on a 0–100 mm VAS); falling Patient-Rated Wrist Evaluation scores at 3-month review; progressive radiographic collapse; or new MRI/arthroscopic evidence of articular surface compromise. A meaningful proportion of patients reach a symptomatic plateau despite radiographic progression [10]; radiographic deterioration alone, without clinical correlate, is therefore not an automatic indication for surgery in the older or paediatric patient.

### 4.8. Psychosocial Factors, Fear-Avoidance, and Self-Efficacy

Prolonged uncertainty, motion ceilings, and occupational threat leave Kienböck patients vulnerable to pain catastrophising and kinesiophobia. The Pain Catastrophising Scale (13 items, 0–52; cut-off ≥ 30) [65], the Tampa Scale for Kinesiophobia (concept [66]; psychometrics [67]; cut-off ≥ 37), and the Pain Self-Efficacy Questionnaire (10 items, 0–60) should be screened at baseline and at 3-month review. PCS and TSK have been validated in hand-surgical cohorts and predict prolonged disability after distal radius and hand-forearm injury [68,69,70,71]. Psychologically informed practice (explicit reassurance about the natural-history nuance of stage I–II disease, graded exposure rather than passive avoidance, and self-efficacy-building activity diaries) should accompany the orthotic and exercise programme. A screen-then-act pathway is therefore recommended: re-administer PCS, TSK and PSEQ at baseline, at the 3-month review point, and at any escalation decision; use population-specific anchors rather than a single threshold. Hand-surgery cohorts predict prolonged disability at PCS ≥ 20 (Roh et al. 2014 [68]; Ozkan et al. 2017 [69]) rather than at the Sullivan et al. 1995 chronic-pain ≥ 30 cut-off [65,66]; PSEQ scores < 40 are conventionally a moderate-confidence marker for self-efficacy concerns (Nicholas et al. 2007 [72]). A tiered response follows: psychologically informed practice (pain neuroscience education and graded exposure rather than passive avoidance) for moderate scores; multidisciplinary referral (CHT-led activity pacing, OT-led occupational counselling, and MD-led consultation-liaison input where indicated) for severe scores or non-response by 3 months. Explicit referral thresholds and a named responsible clinician should be documented in the local protocol. The operational depth above is necessarily abbreviated; the absence of a multidisciplinary CHT-led evidence base in Kienböck rehabilitation remains a clear priority (see Section 8), and the Section 8.3 trial proposals include a PCS/TSK/PSEQ measurement programme as part of the prospective evaluation of this pathway. As a Section 2.4 reflexivity note, the choice of PCS/TSK/PSEQ over PROMIS Anxiety/Depression/Pain Interference reflects the European hand-surgical convention from which the author group draws and the availability of multilingual validated versions; the trade-off against PROMIS-batterie comparability is acknowledged.

**Table 2:** Adjunctive and pharmacological modalities considered in conservative management of Kienböck disease, with evidence summary and recommendation. OCEBM = Oxford Centre for Evidence-Based Medicine 2011 levels; GRADE qualitative certainty. Insufficient evidence to recommend indicates absence of Kienböck-specific prospective data and does not imply established lack of benefit.

Modality	Specific Evidence	Adjacent Evidence	OCEBM Level	GRADE Certainty	Recommendation
Volar wrist orthosis, 6–12 wk, neutral −15° extension	Expert consensus [9,12]	Distal-radius CPG [63]	4–5	Very low	Reasonable expert-consensus trial; weaning rather than open-ended immobilisation
Dart-throwing-motion-plane mobilisation	None Kienböck-specific	DTM kinematics [15,44,45]; hand-therapy protocol [51]	5	Very low	Biomechanically rationalised; outcome data awaited
Sensorimotor/proprioceptive retraining	None	Dorsal-ligament reflex evidence [16,49]	5	Very low	Reasonable adjunct; not first-line
Oedema management (MEM, Coban, contrast baths)	None	Hand-therapy syntheses [59,60,61,62]	4–5	Low	Standard hand-therapy adjunct
NSAIDs for symptom control	None Kienböck-specific	General MSK practice	5	Low	Reasonable for symptoms; no disease-modifying claim
Bisphosphonates (alendronate, pamidronate)	None (no Kienböck RCT)	Femoral-head osteonecrosis only [54]	5	Very low	Insufficient evidence to recommend
Pulsed electromagnetic field (PEMF)	None Kienböck-specific	Non-union historical	5	Very low	Insufficient evidence to recommend
Extracorporeal shock-wave therapy (ESWT)	None Kienböck-specific	Mixed-site bone-marrow-oedema SR [55]	5	Very low	Insufficient evidence to recommend outside research
Hyperbaric oxygen therapy (HBO)	Case report [56]	-	5	Very low	Anecdotal; not recommended outside investigational context
Psychosocial screening (PCS, TSK, PSEQ) at baseline and 3 mo	None Kienböck-specific	Hand-cohort validation [68,69,70,71]	4	Low	Recommended as standard practice

## 5. Procedure-Specific Postoperative Rehabilitation

The published rehabilitation literature after Kienböck surgery is dominated by descriptive single-centre protocols embedded in surgical case series. Of the cohorts cited below, six joint-levelling series report ranges [73,74,75,76,77,78,79]; one reports a standard deviation on follow-up duration [80]; and none report 95% confidence intervals for the principal patient-reported outcome. Hones et al. 2024 [81] and Park et al. 2023 [82] are the only pooled sources with dispersion fit for a quantitative reading. Table 3 and Table 4 collate outcome data; Table 5 and Table 6 collate per-procedure rehabilitation timelines with provenance flagged (P) primary publication, (C) expert consensus, and (A) authors’ practice. The prose below concentrates on rationale, evidence anchors, and rehabilitation-relevant pearls; routine cast and phase durations are not duplicated.

### 5.1. Radial Shortening Osteotomy (RSO)

RSO is the most common joint-levelling procedure for early Kienböck disease. A single-anchor “stable to 20 years” reading is no longer defensible. The Hokkaido school led by Iwasaki has been one of the most consistent producers of long-term RSO data since the mid-1990s, with biomechanical and clinical anchor work that pre-dates and informs the Matsui synthesis cited below. Matsui et al. reported mean follow-up 14.3 years (range 10–21) in 11 wrists with Modified Mayo Wrist Score (MMWS) 92 and no Lichtman progression [77]. Watanabe extended observation to a mean of 21 years in 13 DASH respondents, with grip at 88% of contralateral and MMWS 83 [78]. Werners 20–33-year cohort (*n* = 16) reports grip at 95% of contralateral, DASH 6.1, and Lichtman stability in 56% [8]. Almquist et al. 1982 remains the operative anchor [79]; Luegmair et al. 2017 and Tatebe et al. 2016 complete the long-term picture [75,76].

The dart-throwing-motion (DTM) arc is the first plane mobilised at week 6 because it minimises radiocarpal load and scaphoid-lunate excursion during osteotomy union. Imaging milestones at 3 and 6 months address plate position, union, and the Stahl carpal-height index. Hardware removal at 12–24 months is needed in approximately 10–20% [75]; therapists should look for focal plate tenderness and dorsal-sensory-branch dysaesthesia in the first dorsal web. For ulnar-positive variants, uncommon in Atlantic-rim cohorts but mainstream in the East-Asian literature since the Itoh and Mikami ulnar-shortening osteotomy series, joint-levelling via ulnar shortening offers a parallel option whose rehabilitation timeline approximates the RSO trajectory but diverges in its ulnar-sided dorsal sensory-branch and ECU pathway considerations (the Japanese-language primary literature was not formally searched; cf. Section 2.1 and Section 8.1).

### 5.2. Capitate Shortening Osteotomy (CSO), With or Without Vascularised Graft

The original CSO of Almquist combined capitate shortening with capitohamate fusion to prevent secondary instability [83]. Modern cohorts have abandoned that combination. Gay’s isolated-CSO series (*n* = 18) and Hegazy’s 45-patient quasi-RCT (isolated CSO, ICS, *n* = 21 versus CSO plus 4+5 ECA vascularised graft, CCS, *n* = 24) use neither capitohamate fusion arm [73,84]. Hegazy’s principal finding at a mean of 33 months was a stage-IIIA failure rate of 28% (6/21) with the isolated procedure versus 8% (2/24) with CSO plus VBG, with CCS also superior on visual-analogue pain, ROM, grip, MMWS, and lunate- and carpal-height indices. The biomechanical rationale for the historical capitohamate step is preserved in Viola et al. [85] but is not routine. Yildirim’s median 62-day return to work informs the light-duty timeline [86]. The older intercarpal-fusion motion restrictions no longer apply.

### 5.3. Vascularised Bone Grafting (4+5 ECA, Volar Mathoulin, Pisiform, Medial Femoral Trochlea)

Vascularised bone grafts (VBG) aim to restore lunate vascularity in stage I-IIIA disease with preserved articular cartilage. The dorsal 4+5 extensor compartmental artery graft of Moran and Bishop has the best-anchored medium-term data: in 26 patients at a mean of 31 months, motion was preserved (68% to 71% of contralateral; the operation does not aim to restore range), grip improved from 50% to 89%, 92% reported pain improvement, 85% met Lichtman outcome criteria, and 77% had no further radiographic collapse [87]. Pedicle anatomy is detailed in Sheetz, Bishop and Berger [88]; operative reference: Bishop [89]. Mathoulin’s volar pedicled VBG offers an alternative when the dorsal route is unsuitable [90]. The vascularised pisiform transposition (Saffar procedure) [91] belongs in Lichtman II-III, not salvage: Daecke’s long-term pisiform-reinforcement series reported DASH 15.3 ± 17.9 and Cooney 82.4 ± 10.0 at 12 years in 23 patients, with collapse prevented in 16/22 cases [92,93]. Japanese groups have added a distinct pedicled variant. Matsumoto and colleagues grafted vascularised bone from the distal radius and held the lunate off-load with temporary scaphocapitate fixation for four months; in 26 stage III wrists, this maintained pain relief and carpal height to at least two years [94]. The fixation interval sets the point at which loaded rehabilitation can begin.

The mainstream clinical trajectory has shifted in the past decade from the pisiform transposition lineage toward the medial femoral trochlea (MFT) osteochondral free flap as the procedure of choice in IIIA wrists with Schmitt B viability and poor lunate substance, on chondrocyte-viability and surface-architecture grounds [95,96]; the Daecke long-term pisiform-reinforcement data are retained above as the historical comparator that frames that shift. The medial femoral trochlea (MFT) osteochondral free flap closes the only substantive technique gap. Bürger (*n* = 16, follow-up 19 months) reported union in 15/16 cases, complete pain relief in 12, and grip at 85% of contralateral [97]. Pet (*n* = 18, 2.1 years) reported DASH 10.8, PRWE 18.1, grip 68%, pinch 90%, and carpal height maintained, with the Pet and Higgins groups subsequently extending follow-up and cohort size in the 2023–2024 literature [95,96] while comparative data against pisiform transposition (Daecke et al. 2005 [92,93]) increasingly favour the MFT route in suitable candidates [98]. Higgins provides the technical review and broader osteochondral-free-flap context [95,96]. Knee donor-site morbidity is acceptable: Windhofer reports KOOS subscales 83.1–96.8 at one year with transient deep-flexion discomfort [99]. Pre-operative knee counselling is mandatory. Pooled comparative evidence is in Park et al. 2023 [82], a systematic review of observational cohort studies rather than a pooling of jointly-randomised arms, reports non-operative Lichtman worsening 40.2% (95% CI 25.7–56.6) and VBG 17.0% (95% CI 10.2–26.9); the non-overlapping confidence intervals are consistent with, but do not establish, a between-arms benefit [82] and Tsantes’ VBG-consolidation systematic review [100].

Rehabilitation protects both the recipient lunate and the donor pedicle. Above-elbow plaster for two weeks limits pronosupination that could torque the pedicle. DTM-plane motion starts at week 6; pronation extremes are deferred to week 10. Serial MRI at 6 and 12 months is routine, and strenuous load is deferred until imaging confirms revascularisation. The MFT timeline is slower throughout (above-elbow 0–4 weeks; protected ROM week 8; heavy return 16–24 weeks gated by incorporation), in keeping with osteochondral biology and knee donor-site precautions [95].

### 5.4. Lunate Core Decompression

Lunate core decompression, open via dorsal approach or arthroscopic, is attractive because it does not interfere with surrounding articulations [101,102,103]. The Mehrpour et al. 2011 [101] open cohort (*n* = 20, stages I–IIIB) reported, at five years, a reduction in pain frequency (visual analogue scale) from a mean of 88 to 14 and in DASH from 84 to 14 (both *p* < 0.001), with no decompression-related complications and no carpal collapse (carpal height ratio 0.56 to 0.55). The metaphyseal Illarramendi procedure shares the brief pathway, with Schulz reporting a mean of 5.7 weeks to original occupation [104]. Mehrpour reports 2/20 conversions to radial shortening for persistent pain, which should be discussed pre-operatively. A less-invasive Japanese approach pairs lunate drilling with autologous bone-marrow transfusion, pulsed ultrasound, and short-term external fixation. Ogawa and colleagues reported durable pain relief and functional gain in Lichtman II and III wrists at a mean of six years [105]; here too the external-fixation period governs when axial loading can resume.

### 5.5. Limited Intercarpal Arthrodesis: STT and Scaphocapitate

When the lunate is non-viable but surrounding cartilage is preserved, limited intercarpal arthrodeses bypass the lunate. STT (scaphotrapeziotrapezoidal) fusion was described by Watson [106]; Meier’s series (*n* = 59 of 84) is the largest modern STT cohort [107]. Scaphocapitate (SC) arthrodesis, originally described by Sennwald, is technically more straightforward but shifts compressive load toward the radioscaphoid articulation [108]. Iorio’s mixed SC plus STT-C cohort (*n* = 12, mean 13 months) reported pain reduction from 6.6 to 2.8 on a 10-point scale (W = 18, *p* < 0.05) [109]. Charre’s long-term SC follow-up (*n* = 17, mean 10.7 y, SD 7.1) reports late radioscaphoid degeneration in 2/17 cases at 17–22 years (11.8%, Wilson 95% CI 3.3–34.3%) [80].

STT and SC have divergent loading consequences (Table 5 and Table 6): STT preserves the radiolunate articulation but transfers compressive load onto the radioscaphoid joint, with the DTM plane the most tolerated arc long-term; SC eliminates the scaphoid as a moving body, so loaded dorsiflexion and radial deviation must be limited beyond week 10 to spare the radioscaphoid contact. Patients should be counselled that limited fusion permanently restricts the motion arc to roughly 50–60% of contralateral and that rehabilitation targets stabilising that plateau rather than pursuit of full motion.

### 5.6. Four-Corner Arthrodesis (4CF)

Four-corner (lunate-capitate-hamate-triquetrum) arthrodesis is principally a SLAC/SNAC procedure but is increasingly used in Kienböck stages IIIB-IV when the capitate head requires offload and the lunate is sacrificed [110,111]. The strongest pooled evidence is Hones et al. 2024 [81], a systematic review of observational cohort studies (61 studies, *n* = 3174), reporting 4CF nonunion at 8.9% and revision at 11%, with 95% confidence intervals throughout, extrapolated from the largest contemporary SLAC/SNAC meta-analysis, since no Kienböck-specific 4CF meta-analysis is available [81]. Mulford’s earlier systematic review (52 studies) remains a relevant comparator [111]; Saltzman’s post-traumatic OA systematic review provides context [112].

Rehabilitation differs from PRC: immobilisation is longer (short-arm cast 4–6 weeks pending consolidation, versus 10–14 days for PRC), and the working surface is the midcarpal-DTM arc, with DTM-plane motion introduced from week 6–8 after radiographic consolidation, forearm rotation from week 4, and no loading until weeks 16–24 (Table 5). Dorsal hardware sensitivity is common and may prompt later hardware removal.

### 5.7. Proximal Row Carpectomy (PRC)

PRC removes the scaphoid, lunate, and triquetrum, creating a new articulation between the proximal capitate and the lunate fossa. It is appropriate in Lichtman IIIB and selected IIIC, particularly in patients over 40; in stage IV the proximal capitate cartilage must be assessed intra-operatively with a conversion plan agreed beforehand [113]. Cohen and Kozin’s comparison with 4CF remains foundational [114]. The long-term cohort literature includes Jebson at 10 years [115], Ali at 15 years [116], Wall at 20 years (a cohort study, not a meta-analysis) [117], Stern [118], Croog [119], and Wagner (*n* = 144, mixed-indication PRC cohort with manual-labourer status flagged as outcome variable) [120]. Teunissen’s modern routinely-collected outcomes report median RTW 12 weeks (range 3 weeks to 35 months) [121]; Lin’s current-concepts review consolidates PRC versus 4CF [122]; Wagner’s <45-year comparison is the key age-stratified analysis [123].

Rehabilitation focuses on functional DTM-plane motion rather than on maximisation of arc, because the radiocapitate joint is the new load-bearing surface. Expected motion is 60–70% of contralateral. Ongoing pain or stiffness at 6–12 months should trigger re-imaging for radiocapitate degeneration; Hones’ pooled fusion conversion is 5.2% (extrapolated from the same SLAC/SNAC meta-analysis; no Kienböck-specific source) [81]. Manual labourers carry higher revision risk per Wagner, and pre-operative consent should reflect that.

### 5.8. Total Wrist Arthrodesis (Fusion)

Total wrist arthrodesis is the salvage of choice in Lichtman IV with multi-compartmental arthrosis and in younger high-demand patients in whom motion-preserving options have failed [124,125]. The fusion position is 10–15° dorsiflexion with slight ulnar deviation on the dominant wrist and neutral on the non-dominant; this is the position that maximises grip and tool use [126]. Sauerbier’s Kienböck-specific series reported 70% return to original occupation, including heavy labour, with a DASH of 51.4 [127]. Hastings quantifies the expected ADL limitations (buttoning, perineal hygiene, hammer, screwdriver) [128]; the field corroborates the ADL profile [129]; and Rodríguez-Merchán consolidates the current literature [130]. Compensatory motion training (forearm rotation for tool manipulation, two-handed task strategies) is integral and is best introduced pre-operatively as part of expectation setting.

### 5.9. Wrist Denervation (PIN ± AIN)

Wrist denervation offers symptomatic pain relief without altering articular anatomy. Buck-Gramcko et al. 1977 series is the historical anchor [131]; Berger’s partial denervation refined the technique [132]; the follow-up reports 85% pain relief at 2.5 years [132]; O’Shaughnessy documents a “buys time” effect to a median of 8 years [133]. Chin et al. 2020 [134] systematic review reports return-to-work rates up to 94% (pooled across a mixed-indication SR of cohort studies with heterogeneous attrition; the upper bound should not be read as procedure-typical) and a partial-denervation re-operation rate of 19% [134]; Fidanza finds no clear advantage of combined AIN plus PIN over isolated PIN [135]; and Milone completes the contemporary picture [136].

Two rehabilitation-specific caveats apply. First, denervation does not retard disease progression; re-imaging plans must not be deferred on the basis of symptomatic improvement. Second, AIN-derived afferents contribute to proprioceptive feedback, and partial denervation degrades reflex stabilisation; in high-precision or safety-critical occupations (machine operation, hot work), the loss of pain-warning function must be discussed and documented before clearance.

### 5.10. Total Wrist Arthroplasty (TWA)

Total wrist arthroplasty is appropriate in Lichtman IV pan-carpal disease in lower-demand patients aged over approximately 55 years, in rheumatoid wrists, where the contralateral wrist is fused, and where motion preservation is the patient priority. Yeoh et al. 2015 systematic review remains the pooled-evidence anchor [137]; Boeckstyns and Herzberg provide the complications update [138]. By implant family: the Motec series report 5-to-10-year survivorship at 82% [139,140,141,142]; universal-2 mid- and long-term survivorship 68–78% [143,144,145] and ReMotion 90% at 5–9 years [144,146]. Berber consolidates the motion-preserving salvage rationale [147].

Two principles override the rehabilitation timeline set out in Table 5 and Table 6. First, no passive ROM and no resistance before week 8; gentle active motion from week 2 is pain-led, not target-led. Second, standard wrist flexion-extension is preferred over DTM-axis training in some implants because the DTM axis may compromise component bearings. Institutional protocols vary and must be reconciled with the implanting surgeon before rehabilitation starts (authors’ practice and expert consensus; primary biomechanical evidence on DTM-axis loading of TWA bearings is awaited).

Lifetime activity restrictions after TWA-Arthroplasty are explicit and must be documented before consent. Heavy manual labour is contraindicated; sustained lifting ≈ 5 kg, occasional ≈ 10 kg; impact sport, power-tool use, hand-arm vibration and repetitive loading are contraindicated for the implant lifetime, and bear on SUVA/DGUV and equivalent occupational-medicine assessment [137,138]. The contrast with TWA-Arthrodesis, where heavy labour is the expected outcome, should be made explicit pre-operatively, and the choice between fusion and arthroplasty framed as a motion-versus-load trade-off.

**Table 3:** Comparative procedure outcomes: cohort size and evidence base.

Procedure	*n* Studies	*n* Patients (Total)	Mean FU	Dispersion in Source?	Evidence Grade
RSO (Radial Shortening Osteotomy)	7 cohorts	~95–110	14–25 y (long-term subset)	Ranges only; no CIs/SDs	Low (point estimates, *n* < 20 per study)
CSO ± VBG (Capitate Shortening Osteotomy)	3 (1 RCT-design)	45 + 18 + 35	33 mo (Hegazy); 62 d post-op median RTW (Yildirim)	Between-group *p*-values (Hegazy); ranges (Yildirim)	Low-Moderate (Hegazy quasi-RCT)
VBG:4+5 ECA (Moran-type)	1 cohort	26	31 mo	None (full text reports Stahl/CHI)	Low
VBG: Mathoulin volar	1 cohort	22	≥5 y	None	Low (small cohort)
VBG: Pisiform (Daecke long-term reinforcement)	1 cohort	23 (20 with pre-op x-rays)	12 y	SDs reported (DASH, Cooney)	Low-Moderate (long FU + SDs)
MFT free flap (Medial Femoral Trochlea)	2 main cohorts + reviews	16 + 18 + 2 reviews	1.4–2.1 y	None in main cohorts	Low (*n* = 16, *n* = 18)
STT arthrodesis	1 large cohort + classic	59/84 (Meier) + classic Watson	4 y (range 2–8); long-term 22 y (Charre)	None reported	Low
SC arthrodesis	4 cohorts	11 (Sennwald) + 12 (Iorio) + 17 (Charre) + Vahedi	1.1 y (Iorio) to 10.7 y (Charre, SD 7.1)	SD on FU (Charre); statistical *p* < 0.05 (Iorio)	Low-Moderate (Charre)
Lunate core decompression	1 open + emerging arthroscopic	20 (Mehrpour 2011 [101])	5 y	None	Very low (*n* = 20, single center)
Illarramendi/metaphyseal core decompression	1 cohort	small	several years	n/r	Low
PRC (Proximal Row Carpectomy)	5+ cohorts + 2 large MA	*n* = 3174 (Hones et al. 2024 [81] pooled)	10–20 y (Wall, Ali, Wagner)	95% CIs in Hones et al. 2024 [81] MA	Moderate (large MA, Hones et al. 2024 [81])
4CF (Four-Corner Arthrodesis)	5+ cohorts + Hones MA	*n* = 3174 pooled (Hones)	10 y	95% CIs in Hones et al. 2024 [81] MA	Moderate
TWA-Arthrodesis (Fusion)	4+ cohorts	23 (Hastings); Sauerbier Kienböck-specific	varies	None reported as CIs	Low (point estimates, small *n*)
TWA-Arthroplasty	Multiple per implant family	1000+ across registries	5–22 y	Reigstad revision survivorship with CIs	Moderate (Yeoh et al. 2015 [137] SR + Meulendijks et al. 2024 [148] + Hones et al. 2024 [81])
Wrist Denervation (PIN ± AIN)	Chin et al. 2020 [134] SR + Fidanza et al. 2023 [135] MA + classics	*n* > 500 pooled across SRs	up to 8 y	Chin et al. 2020 [134] SR has pooled data with CIs	Low-Moderate

**Table 4:** Comparative procedure outcomes: clinical and radiographic results.

Procedure	Grip (% Contra)	DASH/QuickDASH	MMWS (Mean)	VAS Pain (Post)	RTW Weeks (Light/Heavy)	Lichtman Progression
RSO (Radial Shortening Osteotomy)	88 (Watanabe)	DASH 5–12 (range)	79–92	n/r	6–8/13–26 wk (consensus)	0–6/12 (range across cohorts)
CSO ± VBG (Capitate Shortening Osteotomy)	improved (Hegazy stage IIIA)	MMWS improved (Hegazy)	n/r as primary	not enumerated by stage	~9/not reported (Yildirim et al. 2020 [86])	ICS 28% (6/21) vs. CCS 8% (2/24) at stage IIIA (Hegazy)
VBG:4+5 ECA (Moran-type)	50 pre to 89 post	n/r (Lichtman score 85% satisfactory)	n/r	improved in 92%	8 wk light/4–6 mo heavy (paper-protocol)	no further collapse in 77%
VBG: Mathoulin volar	n/r	n/r	n/r	n/r	n/r	n/r
VBG: Pisiform (Daecke long-term reinforcement)	84	DASH 15.3 ± 17.9	Cooney 82.4 ± 10.0	reduced (n/r single number)	n/r	stage unchanged 11, improved 3, progressed 6 of 20; 7/22 OA at FU
MFT free flap (Medial Femoral Trochlea)	68 (Pet); 85 (Bürger)	DASH 10.8 (Pet); n/r in Bürger	n/r	improved (12/16 complete relief, Bürger)	n/r	unchanged 10, improved 4, worsened 2 (Bürger *n* = 16)
STT arthrodesis	NR	n/r	n/r	n/r	10–14/24+ (consensus)	n/r
SC arthrodesis	not enumerated	n/r	n/r	6.6 → 2.8 on 10-point scale (Iorio et al. 2015 [109])	24 (full) per Hegazy et al. 2024 [149]	n/r in main cohorts; long-term radioscaphoid degeneration 2/17 at 17–22 y (Charre)
Lunate core decompression	not enumerated	84 → 14	n/r	88 → 14	4–6/12 (Section 5.4)	none in 18/20
Illarramendi/metaphyseal core decompression	not enumerated	n/r	n/r	reduced	mean 5.7 wk to original occupation; all returned	n/r
PRC (Proximal Row Carpectomy)	65–80 (consensus)	DASH 15–25	n/r as primary	1–3/10	median 12 (Teunissen)/24+ for heavy	Conversion to fusion 5.2% (Hones); 12% @ 13 y (Wagner)
4CF (Four-Corner Arthrodesis)	65–75 (consensus)	DASH 18–28	n/r	1–3/10	~10/24+ (mixed SLAC/SNAC cohorts; not Kienböck-stratified)	nonunion 8.9% (Hones MA); revision 11%
TWA-Arthrodesis (Fusion)	70–85 (lever arm preserved)	DASH 30–50; Sauerbier 51.4	0 ROM by definition	1–2/10	12/16–26 (heavy-trade feasible, Hastings)	n/a (endpoint)
TWA-Arthroplasty	60–80	DASH 20–35	n/r as primary	1–3/10	12 (light only); HEAVY CONTRAINDICATED: lifetime restriction ~5 kg/no impact/no power tools	5–9 y implant survival 70–82% (Motec 82%, ReMotion 90% @ 5–9 y, U2 68–78%)
Wrist Denervation (PIN ± AIN)	7–64% gain reported	DASH 15–30	n/r	36–92% reduction	2–6/2–6 wk	n/r as primary outcome

**Table 5:** Per-procedure rehabilitation timeline (provenance-flagged): immobilisation and motion progression. Flags follow the outline of Section 5: (P) primary publication, (C) expert consensus, (A) authors’ practice. Intervals marked (C) or (A) are not standards of care and should be individualized.

Procedure	Cast/Immobilisation	Splint/Orthosis Transition	ROM Start	Strengthening Start	DTM-Plane Priority
RSO	Above-elbow plaster w/thumb spica 0–2 wk (Matsui); OR forearm cast (Almquist et al. 1982 [79])	Short-arm cast wk 2–6; removable thermoplastic orthosis wk 6–8	Wk 6 active wrist motion (DTM-plane primary); digits/shoulder/elbow from d1	Wk 10–14 (isometric grip; forearm rotation)	High (radiocarpal protection during union)
CSO isolated	Short-arm cast 0–4 wk (Gay et al. 2009 [84], Hegazy et al. 2021 [73] ICS)	Removable wk 4–8	Wk 4 (intercarpal union faster than metaphyseal)	Wk 8–12	High
CSO + VBG (4+5 ECA)	Short-arm cast 0–6 wk (Hegazy CCS)	Removable wk 6–10	Wk 6	Wk 10–14	High
VBG:4+5 ECA (Moran)	Above-elbow 0–2 wk	Short-arm cast wk 2–6; thermoplastic wk 6+	Wk 6 (DTM-plane active)	Wk 10 (pedicle protection: avoid pronation extremes)	High
VBG:Volar Mathoulin	Above-elbow 0–2 wk	Short-arm wk 2–6	Wk 6 DTM-plane	Wk 10	High
VBG:Pisiform (Saffar/Daecke)	Short-arm 0–6 wk; pisiform pedicle protection	Removable orthosis wk 6–12	Wk 6 cast-out weeks	Wk 12 grip	High
MFT free flap	Above-elbow 0–4 wk (Curtis/Klagenfurt; Higgins et al. 2017 [95])	Short-arm cast wk 4–8; orthosis with protected active F/E wk 8–12	Wk 8 protected; Wk 12 progressive	Wk 12 grip	High
Lunate core decompression (Mehrpour open)	Removable orthosis 0–2 wk (comfort)	n/a (no rigid immobilisation)	Wk 0 active digits + wk 2 wrist	Wk 6 (pain-led)	Medium
Illarramendi/metaphyseal CD	Short-arm cast 4 wk	Removable 4–8 wk	Wk 4	Wk 6	Medium-High
STT arthrodesis	Short-arm thumb-spica cast 0–6 wk	Removable thumb-spica wk 6–10; protected active motion (DTM-plane most comfortable)	Wk 6	Wk 10–14 (gentle radial deviation; STT-plate extension may overload)	High (DTM-plane is the most tolerated arc)
SC arthrodesis	Short-arm thumb-spica OR short-arm cast 0–6 wk	Removable wk 6–10; protected active motion. Differs from STT: radioscaphoid loading INCREASED → limit loaded dorsiflexion beyond wk 10	Wk 6	Wk 10–14 (protected radial deviation; sensorimotor work)	High
PRC	Bulky soft dressing + short-arm splint 10–14 d	Removable thermoplastic between exercises + nightly to wk 8–12	Wk 2–3 AROM; Wk 4–6 PROM	Wk 6–8 (no loading/no power grip until wk 16–24)	Medium-High (radiocarpal arc is the new working surface)
4CF	Short-arm cast 4–6 wk (bone-union dependent)	Removable to wk 8–10 (consolidation awaited)	Wk 6–8 (after radiographic consolidation); pronation/supination from wk 4	Wk 10–12 (no loading until wk 16–24)	High (DTM-plane is the natural midcarpal arc)
TWA-Arthrodesis (Fusion)	Short-arm splint 0–2 wk → cast or functional brace 6 wk to radiographic union	Brace/cast	Digits/thumb/elbow immediate; wrist 0° by definition	Wk 8 after consolidation	n/a (no wrist motion)
TWA-Arthroplasty	Bulky dressing + volar/dorsal splint 1–2 wk	Removable wk 1–6/8	Wk 2 active wrist (gentle, pain-led)	NO passive ROM, NO resistance before wk 8	Medium-Low (DTM-axis may compromise component bearings; institutional protocols vary)
Wrist Denervation (PIN ± AIN)	Minimal dressing; load from d1–3 pain-adapted	Splint optional 7–10 d comfort	AROM/PROM immediate	Wk 2–3 grip work	Medium

**Table 6:** Per-procedure rehabilitation timeline (provenance-flagged): return to work and procedure-specific considerations. Provenance flags (P, C, A) as defined for Table 5.

Procedure	RTW Light	RTW Heavy	Procedure-Specific Notes
RSO	6–8 wk sedentary; 3 mo light manual (C)	4–6 mo (P consensus across long-term cohorts)	Imaging milestones 3 + 6 mo; hardware removal 12–24 mo if symptomatic (~10–20% per Luegmair et al. 2017 [75])
CSO isolated	6 wk	3–4 mo	CH fusion NOT routine in modern cohorts (Gay et al. 2009 [84], Hegazy et al. 2021 [73])
CSO + VBG (4+5 ECA)	8 wk	4–6 mo	MRI revascularisation 6–12 mo (P); failure 8% vs. 28% isolated CSO stage IIIA (Hegazy et al. 2021 [73])
VBG:4+5 ECA (Moran)	6–8 wk	4–6 mo (after MRI revascularisation)	Surveillance MRI 6 + 12 mo (Moran et al. 2005 [87]); revasc ~71% imaged
VBG:Volar Mathoulin	8 wk	4–6 mo	Protect dorsal/volar sensory branches and wound before resistive exercise
VBG:Pisiform (Saffar/Daecke)	6–8 wk	4–6 mo (gated by MRI 6–12 mo)	Concurrent radial shortening in ulnar-minus patients; protect that construct
MFT free flap	8–12 wk	16–24 wk; high-impact restricted until radiographic incorporation 3–4 mo	Knee donor: Windhofer et al. 2016 [99], partial weight-bearing crutches 2 wk; KOOS ≥ 80% at 1 y; transient anterior knee discomfort with deep flexion. Counselling mandatory.
Lunate core decompression (Mehrpour open)	4–6 wk	12 wk (pain-led)	2/20 conversions to RSO if persistent pain (Mehrpour et al. 2011 [101])
Illarramendi/metaphyseal CD	Mean 5.7 wk to original occupation (Schulz et al. 1998 [104])	12 wk (variable)	Single-author cohort; all returned to previous occupation
STT arthrodesis	8 wk	4–6 mo	Watson et al 1985 [106]; Meier et al. 2004 [107]
SC arthrodesis	8 wk; 24 wk full RTW per Hegazy et al. 2024 [149]	4–6 mo	Long-term radioscaphoid degeneration 2/17 at 17–22 y (Charre et al. 2018 [80])
PRC	~3 mo (median 12 wk per Teunissen et al. 2024 [121])	~6 mo; outcomes worse in manual labourers (Wagner et al. 2016 [120])	Full rehab to 12 mo
4CF	~3 mo	~6 mo	Nonunion 8.9% pooled (Hones et al. 2024 [81]); 11% revision pooled
TWA-Arthrodesis (Fusion)	2–3 mo	4–6 mo, heavy-labour explicitly allowed (Sauerbier et al. 2000 [127] 70% RTW to original occupation)	Fusion position 10–15° dorsiflexion + slight ulnar deviation for dominant; ADL limitations: buttoning, perineal hygiene, hammer, screwdriver (Field et al. 1996 [129])
TWA-Arthroplasty	12 wk light only	Heavy manual labour lifetime restricted	Lifetime restriction (Boeckstyns et al. 2024 [138] + Yeoh et al. 2015 [137]): no heavy lifting (>5 kg sustained/>10 kg occasional); no impact sport; no power tools; no repetitive load
Wrist Denervation (PIN ± AIN)	1–2 wk	4–6 wk	Pain-warning-function loss:patient must be counselled; high-precision/safety occupations need special caution. Re-imaging plan with RTW clearance

### 5.11. Emerging Procedures

Several developments fall outside the stage-procedure cells of Section 5.1, Section 5.2, Section 5.3, Section 5.4, Section 5.5, Section 5.6, Section 5.7, Section 5.8, Section 5.9 and Section 5.10 but are visible in contemporary practice and should be acknowledged for completeness. Patient-specific instrumentation and 3D-printed total or partial lunate implants are now used in selected European hand-surgery centres for Lichtman IIIB-IIIC wrists, with rehabilitation timelines extrapolated from the TWA-arthroplasty trajectory rather than from primary data. An arthroscopic-minimisation trend extends the open core decompression of Section 5.4 [43,103] toward arthroscopic-assisted four-corner arthrodesis and proximal-row carpectomy in selected cohorts; rehabilitation differences from the open equivalents are at present surgeon-stated rather than evidence-derived. Capitate resurfacing has been described as a motion-sparing salvage in selected Lichtman IIIB wrists, and pyrocarbon lunate interposition (APSI/Amandys lineage in European practice) continues to be used in salvage decision-making, particularly in continental Europe; both procedures have generated case-series-level evidence only, and neither has procedure-specific rehabilitation evidence in Kienböck cohorts. The appropriate framing for the cluster is “emerging procedures, not yet supported by rehabilitation-specific evidence”; the prospective evaluation of any of these procedures should follow the framework articulated in Section 8. Autologous soft-tissue interposition predates the pyrocarbon and silicone options and still merits mention: Ueba and colleagues excised the lunate and filled the defect with a coiled tendon-ball implant under temporary distraction, reporting mostly excellent or good outcomes at a mean of sixteen years [150]. Few interposition materials carry comparable long-term data.

## 6. Functional Outcome Assessment

Outcome measurement in Kienböck disease is constrained by two structural realities: small, heterogeneous cohorts that limit statistical pooling, and a complete absence of disease-specific instrument validation. No patient-reported outcome measure (PROM) has been validated specifically in a Kienböck cohort, and no anchor-based minimal clinically important difference (MCID) has been derived in this population [151,152]. All thresholds reported below are therefore extrapolated from adjacent pathologies, predominantly distal radius and mixed upper-extremity musculoskeletal cohorts and should be interpreted with that caveat. Table 7 summarises the recommended assessment battery.

### 6.1. Patient-Reported Instruments

The Patient-Rated Wrist Evaluation (PRWE) is the preferred primary PROM: it is wrist-specific, comprises five pain and ten function items, and has the strongest psychometric base in adjacent wrist pathology [17,153]. The MCID of 11.5 points derives from a 102-patient distal-radius cohort [154]; the closest construct-validity evidence in a wrist-OA cohort (*n* = 50) supports its use but does not establish a Kienböck-specific anchor [151]. The Patient-Rated Wrist/Hand Evaluation (PRWHE) extends the PRWE with five finger/thumb items and should be selected only when digit involvement is relevant [155,156].

The Disabilities of the Arm, Shoulder and Hand (DASH) and its 11-item short form QuickDASH provide cross-pathology benchmarking [18,19]. Anchor-based MCIDs of 10.8 points (DASH) and 15.9 points (QuickDASH) from a mixed upper-extremity cohort [157] are corroborated by the most recent systematic synthesis (12–14 and 12–15 points, respectively) [157,158]. Pain should be co-captured by a Numeric Rating Scale (NRS, 0–10) at rest and on provocative loading; NRS is preferred to the Visual Analogue Scale for compliance and equivalence [159,160]. The Patient-Specific Functional Scale (PSFS) covers the requirement for individualised activity outcomes [161,162], and the Michigan Hand Questionnaire (MHQ) is an optional secondary instrument when hand-function granularity is required [163,164].

The Modified Mayo Wrist Score (MMWS) and the Krimmer Wrist Score remain prevalent in older Kienböck case series, but they are clinician-rated demerit composites with no published anchor-based MCID, dichotomised category bias and weak responsiveness [127,152,165]. Both instruments should therefore be reported only as historical comparators, not as primary endpoints.

The Patient-Reported Outcomes Measurement Information System Upper Extremity (PROMIS-UE) v2.0, administered as a computer-adaptive test (CAT), is the contemporary methodological counterpoint to the PRWE/DASH lineage and is recommended here as a co-primary or secondary PROM. PROMIS-UE CAT typically reduces response burden to approximately 4–6 items per administration while showing lower floor and ceiling effects than the DASH and QuickDASH in distal-radius and wrist-osteoarthritis cohorts; a distribution-based MCID of approximately half a standard deviation (on the order of 5 T-score points) and the convergent-validity evidence with the QuickDASH supply the operational anchors [19]. PROMIS-UE is the upper-limb instrument adopted in the ICHOM Hand and Wrist Standard Set [166], and we therefore recommend it as the lowest-burden longitudinal-trajectory mechanism for prospective Kienböck cohorts, retaining PRWE as the primary on convergent-validity and historical-comparison grounds. PROMIS-UE has not been validated in a Kienböck-specific cohort; the MCID quoted above is extrapolated from the wider hand-surgery population and inherits the same caveat that applies to PRWE and DASH/QuickDASH.

### 6.2. Performance-Based Measurement

Grip strength is the dominant performance measure in Kienböck cohorts and should be acquired in conformity with the American Society of Hand Therapists (ASHT) protocol [167]: seated patient, shoulder adducted, elbow flexed to 90°, forearm neutral, wrist between 0 and 30° extension and 0 and 15° ulnar deviation; and Jamar hydraulic dynamometer at handle position II, with three trials per side with at least 15 s inter-trial rest; the three-trial mean is recorded with population-norm referencing [168,169]. Pinch strength (tip, key, three-jaw) using a calibrated B&L gauge and isolated forearm pronation/supination strength should be documented separately, as Kienböck salvage procedures differentially affect these load vectors.

Active wrist range of motion is measured by goniometry across flexion/extension, radial/ulnar deviation and forearm rotation, expressed as percentage of contralateral side. The volar- and dorsal-aligned multicentre protocol [170] is the appropriate reference standard; intrarater reliability exceeds interrater, so a single rater per timepoint should be retained [171]. Dart-throwing-motion plane recording (radial extension to ulnar flexion) is desirable but requires explicit axis definition. The Sollerman test may be reported when an activities-of-daily-living endpoint is mandated [172]; the Jebsen-Taylor test is not recommended in Kienböck, as it was calibrated for neuro-rehabilitation populations and is insensitive to wrist-pain-mediated functional limits [173].

### 6.3. Occupational, Health-Related and Psychosocial Domains

Occupational outcome must be captured beyond first return-to-work. The Work Productivity and Activity Impairment questionnaire (WPAI) captures sustained-RTW elevation across absenteeism, presenteeism and activity impairment [174]; the Work Ability Index is an optional categorical adjunct [175]. The criterion-based clearance algorithm and the grip-threshold harmonisation rationale are developed in Section 7.3. Generic health-related quality of life is best captured by the SF-36 Role-Physical subscale, restricted to that subscale to avoid multiplicity inflation [176]. Psychosocial outcomes (Pain Catastrophizing Scale, Tampa Scale for Kinesiophobia (TSK-17) and Pain Self-Efficacy Questionnaire) should be co-administered with the Section 4.8 psychosocial protocol [65,66,67,72]; hand- and wrist-specific psychometric support is now available [68,69,70,71,177].

### 6.4. Gaps and Prospective Standardisation

No Kienböck-specific MCID, no Kienböck construct-validity study, no completed Core Outcome Measures in Effectiveness Trials (COMET) set for Kienböck or wrist osteoarthritis, and no validated wrist-specific functional movement screen presently exist [178,179]. The ICHOM Hand & Wrist Standard Set, although broader than Kienböck, is the closest contemporary framework reference and is recommended as the cross-walk anchor for future trials [166]; closed-chain upper-extremity tests (CKCUEST, YBT-UQ) cannot be repurposed without violating Kienböck loading constraints [180,181]. These gaps are revisited as concrete research targets in Section 8.2; the most recent current-concepts synthesis [5] reinforces this case, while translational signals on local mesenchymal stem-cell therapy are addressed as an emerging biological intervention in Section 5.11.

A short ICHOM Hand and Wrist cross-walk clarifies where the recommended Kienböck battery aligns with the contemporary outcomes standard and where it deviates from it [166]. The ICHOM set adopts PROMIS-UE CAT (recommended above; convergent with the Section 6.1 recommendation), the EQ-5D-5L for generic health-related quality of life, the Brief Michigan Hand Questionnaire (Brief MHQ) for hand-specific function, single-item pain on a 0–10 numeric scale (concordant with the Section 6.1 NRS recommendation), and a structured work-status field operationalised against the WPAI in Section 6.3. The battery proposed here therefore aligns on PROMIS-UE, NRS pain and the work-status concept, but deviates in three places: (i) we retain PRWE as primary on cross-historical comparison grounds, with PROMIS-UE CAT as co-primary; (ii) we recommend SF-36 Role Physical rather than EQ-5D-5L for the historical wrist-OA-comparison rationale articulated in Section 6.3, and acknowledge that an EQ-5D-5L would be more compatible with cost-utility and ICHOM downstream use; (iii) we recommend the longer MHQ over the Brief MHQ for digit-involvement granularity, while flagging that the Brief MHQ is the lower-burden ICHOM choice. These deviations are reasoned but should be reconsidered in any Kienböck-specific COMET process, where the patient-priority axis (cf. Section 7) would be elicited rather than inherited.

**Table 7:** Recommended Kienböck outcome battery.

PROM/Measure	Domain	Items/Range	Direction	MCID (Cohort, Source)	Kienböck-Validated?	Wrist-Specific?	Recommended Tier
PRWE	Pain + function	15 items, 0–100	↓ better	11.5 (DRF, [154])	No (closest: wrist-OA, [151])	Yes	Primary
PRWHE	+finger/thumb	15 + 5 items	↓ better	None Kienböck-specific; inherit PRWE caveat	No	Yes	Conditional primary
QuickDASH	Upper limb	11 items, 0–100	↓ better	15.9 [157]; 12–15 [158]	No	No	Secondary
DASH	Upper limb	30 items, 0–100	↓ better	10.8 [157]; 12–14 [158]	No	No	Alternative secondary
NRS pain	Pain	0–10	↓ better	≈1.5–2 [159]	n/a	n/a	Co-primary (pain)
PSFS	Patient-specific function	3–5 items × 0–10	↑ better	≈2 [162]	No	n/a	Adjunct
MHQ	Hand function	37 items/6 subscales	↑ better	Per subscale [164]	No	Hand	Optional secondary
MMWS (Cooney et al. 1987 [165] grid)	Clinician composite	100 pts	↑ better	None published	No	Yes	Historical comparator only
Krimmer Wrist Score	Clinician composite	100 pts	↑ better	None published	No	Yes	Optional (German continuity)
Grip (Jamar pos. II, ASHT)	Strength	kg + % contralateral	↑ better	n/a (norms-referenced [169])	n/a	Wrist load	Performance core
Pinch (B&L gauge)	Strength	kg	↑ better	n/a	n/a	n/a	Performance adjunct
Goniometry	ROM (F/E, RD/UD, P/S)	degrees + % contralateral	↑ better	n/a [170,171]	n/a	Yes	Performance core
Sollerman HFT	ADL performance	20 tasks, 0–80	↑ better	n/a	No	No	Optional
Jebsen-Taylor HFT	Timed performance	7 subtests	↓ better	n/a	No	No	Not recommended
SF-36 (Role-Physical)	HRQoL	subscale	↑ better	5–10 (context-dependent, [176])	No	No	Optional
WPAI	Work productivity	6 items/4 domains	↓ better	n/r	No	n/a	RTW core (cross-ref Section 7.3)
WAI	Work ability	7 items/7–49	↑ better	Categorical	No	n/a	Optional
PCS/TSK-17/PSEQ	Psychosocial	per instrument	varies	Cutoffs (PCS ≥ 30; TSK ≥ 37)	Hand validation [70,71]	n/a	Section 4.8 core
ICHOM Hand & Wrist Set	Composite framework	Set of validated instruments	n/a	n/a	Not Kienböck-specific	Yes	Framework reference (cross-ref Section 8.2)

All MCID values are extrapolated from non-Kienböck cohorts. Development of Kienböck-specific MCIDs and a COMET-compliant core outcome set is identified as a research priority (Section 8.2) [178,179]. (↑ = a higher score indicates a better outcome; ↓ = a lower score indicates a better outcome. For PCS and TSK-17, lower scores are better; for PSEQ, higher scores are better.)

## 7. An Integrative Rehabilitation Reference Frame for Kienböck’s Disease

Drawing on the integrated osseous-perfusion-articular model set out in Section 3 and the procedure-specific descriptions of Section 5, we bring existing stage- and procedure-related rehabilitation guidance together into a single Kienböck-oriented reference frame. We do not claim methodological novelty; we combine three already-published axes that have so far been treated separately: osseous staging [4], articular-cartilage staging [43], and the tissue phase-of-healing model that underpins hand-therapy practice [182]. Lichtman, Pientka and Bain combined the first two axes on the surgical side of the consultation [6]; on the rehabilitation side that integration has not previously been made explicit. The closest Kienböck-specific antecedent on the therapy side is the systematic review by Innes and Strauch, which collated treatment evidence across early and late disease without supplying a stage-mapped therapy protocol [13]. The cross-walk in Table 8 makes the antecedent of every element of the reference frame visible.

### 7.1. The Three Axes We Integrate

The osseous axis is taken from Lichtman’s state-of-the-art treatment algorithm, in which stage-by-stage decisions follow radiographic staging [4]; the integrated three-axis update of that algorithm informs procedure selection at the surgical-therapy interface [6]. The articular axis is taken from Bain and Durrant’s articular-pathology model, which sets out vascular, osseous, and chondral phases of disease and is conceptually closer to a phase logic than any other Kienböck-specific source [43]. The tissue phase-of-healing axis is the standard hand-therapy backbone (inflammatory, proliferative, remodelling) codified in Skirven’s textbook, with criterion-based progression analogues drawn from the APTA distal-radius clinical practice guideline [57,63]. Phase architecture and proprioceptive sub-systems are further informed by scapholunate-rehabilitation evidence [16,182]. Watson and Ballet’s classification is cited here only as a surgical SLAC staging analogue, not as a rehabilitation template [183]; where a rheumatoid-hand decision-tree analogue is invoked, we cite Chung and Kotsis [184].

### 7.2. Phase Architecture and Modifying Factors

We retain the three-phase architecture used in the literature we integrate, namely Acute Protection, Recovery, and Return-to-Function, recognising that these are the conventional Skirven-aligned phases rather than a contribution of this review [57]. Within each phase, three modifiers shift the trajectory. Disease stage and lunate viability, taken from the osseous and articular axes, lengthen protection phases and lower the realistic ceiling of functional outcome as disease advances [4,43]. The procedure performed (joint-levelling, vascularised bone grafting, lunate bypass, or salvage) sets the biological clock for fracture, osteotomy, fusion, or graft consolidation. Patient factors (occupational demand, age, dominance, comorbidities, motivation) determine pacing, with paediatric patients deserving particular caution given their greater capacity for biological remodelling and the longer conservative trajectories often appropriate in that group. Two further modifiers sit outside anything a stage table can hold. Intraoperative findings, bone quality, and the stability of fixation achieved at surgery often weigh more heavily on early loading decisions than the preoperative stage does, and only the operating surgeon can grade them. The frame is therefore best read as a set of guiding principles rather than a protocol to be followed step by step. It presumes continuing dialogue between the hand surgeon and the rehabilitation specialist, and a plan built around the individual patient will usually serve better than strict adherence to any predefined algorithm.

### 7.3. Limitations of This Reference Frame

This cross-walk is a clinically reasoned synthesis, not a validated instrument. No prospective Kienböck-specific cohort has been used to derive or test the stage-to-phase mappings set out above; the only Kienböck-specific therapy-side antecedent in the literature is a systematic review that collates evidence rather than a stage-adapted protocol [13]. The stage-specific progression criteria implied by the cross-walk are extrapolated from cross-disease evidence (the APTA distal-radius clinical practice guideline, scapholunate rehabilitation, and criterion-based return-to-sport principles) and have not been validated in Kienböck cohorts [63,182,185,186]. No Kienböck-specific functional movement screen exists; the closest validated upper-extremity batteries were developed for shoulder and distal-radius populations and impose closed-chain wrist loading that may be unsuitable for early-stage disease or during bone-healing windows after joint-levelling or vascularised bone grafting. We therefore identify the cross-walk in Table 8, and the stage-specific cutoff hypotheses it implies, as testable propositions rather than validated thresholds; their prospective evaluation in a Kienböck cohort is set out as a research priority in Section 8.

## 8. Gaps in Evidence and Future Directions

### 8.1. Limitations of the Present Synthesis

Several limitations should be acknowledged. First, the evidence base is limited: almost every rehabilitation recommendation rests on retrospective single-centre case series whose primary purpose was surgical outcomes reporting; randomised trials of rehabilitation in Kienböck disease remain absent [5]. We applied the ROBINS-I tool [27] to each contributing cohort.

Second, no Kienböck-specific core outcome set has yet been developed; a COMET-style consensus process with patient and public involvement [179] would strengthen future outcome selection and remains a priority for future work.

Third, this is a narrative review, and language eligibility was restricted to English, German, French, Italian and Spanish; the Japanese, Chinese and Korean literature was not systematically searched, which may under-represent East-Asian contributions to joint-levelling and motion-based rehabilitation.

Finally, the integrative reference frame proposed in Section 7 is a clinically reasoned synthesis rather than a validated instrument and requires prospective evaluation; the trials outlined in Section 8.3 address the principal evidence gaps identified here.

### 8.2. Heterogeneity of Outcome Reporting

Outcome instrumentation is inconsistent: MMWS, DASH or QuickDASH, the PRWE [153], and narrative description are all in routine use, and MCIDs are routinely transposed from distal radius cohorts [154] without caveat. The field needs a Kienböck-specific core outcome set developed via COMET methodology [178,179] and aligned with the ICHOM Hand and Wrist set [166]. As a working minimum: PRWE; QuickDASH; bilateral grip as percentage of contralateral, measured to ASHT positioning; three-plane range of motion including the dart-throwing arc; sustained return-to-work at 6 and 12 months; and standardised imaging at 6 and 12 months for biology-protecting procedures.

### 8.3. Four Trials That Would Change Practice

Trial 1: Early dart-throwing-plane mobilisation after radial shortening osteotomy. Population: Lichtman II-IIIA adults undergoing RSO [6]. Intervention: structured DTM-plane mobilisation from week two [47,187]. Comparator: conventional anatomic-plane mobilisation. Primary outcome: PRWE at 6 and 12 months. Design: parallel two-arm RCT, eight to ten hand centres, ~150 patients.

Trial 2: Structured sensorimotor retraining after vascularised bone grafting. Population: adults undergoing 4+5 ECA VBG [82]. Intervention: eight-week protocol with joint-position-sense retraining, conscious co-contraction, and surface electromyographic biofeedback of FCR/FCU/ECU [16,49]. Comparator: standard postoperative therapy. Primary outcome: PRWE at 12 months. Design: cluster-randomised at site level; ~12 sites, ~200 patients. Cluster design-effect: with assumed intra-cluster correlation 0.03–0.08 and an average cluster size of ≈16 per site, the design effect is 1.5–2.3, inflating the per-arm effective n requirement from ≈64 to roughly 100–150; the ≈200-patient target should accordingly be regarded as a lower bound.

Trial 3: Conservative versus surgical management in Lichtman IIIA, long-term. Population: adults newly diagnosed at IIIA with ulnar-neutral or negative variance. Intervention: structured 12-month conservative programme (immobilisation, unloading orthosis, DTM-plane therapy, optional bisphosphonate). Comparator: indication-matched joint-levelling osteotomy. Primary outcomes: PRWE at 5 years; progression to stage IV; secondary surgery; sustained return-to-work. Design: multicentre RCT with 10-year follow-up; ~240 patients (≥60/arm to detect a 12-point PRWE difference at 5 years assuming SD 22 and 25% attrition).

Trial 4: Sustained return-to-work after Kienböck surgery: a pan-European prospective register. Population: consecutive adults undergoing any Kienböck-indicated procedure, including PRC, four-corner fusion, and total wrist arthrodesis (capturing the salvage stratum). Exposure: procedure type with ISCO-08 occupation coding and Dictionary of Occupational Titles physical-demand stratification [188]. Primary outcome: sustained return to original occupation at 6 and 12 months, capturing occupational downgrade and retraining (WPAI [174]; Work Ability Index [175]; ICHOM work-status field [166]). Design: prospective cohort, ~800 patients across 20 sites over three years. This register would close the single most consistent data gap across the reviewed literature: every cited cohort reports first return-to-work; none reports sustained employment at 12 months.

### 8.4. What This Paper Should Be Worth in Five Years

A reasonable question of any review is its residual value once the next generation of primary studies appears. If the trials above are launched, this paper should age into an artefact: a 2026 snapshot taken before the DTM-plane, sensorimotor, conservative-versus-surgical IIIA, and sustained return-to-work questions had been answered. If they are not launched, it will continue to be cited as a substitute for evidence that does not yet exist. The most defensible use of this synthesis is therefore as a scaffold for trial design: a stocktake of what is known, a tabulation of what is not, and a set of registered, powered, COMET-aligned and CHT-co-authored proposals against which to plan the next decade of Kienböck rehabilitation research.

## 9. Conclusions

Rehabilitation in Kienböck disease occupies a curious position in the literature: clinically central yet bibliographically thin, sustained largely by extrapolations from adjacent wrist conditions and by inherited expert opinion rather than by procedure-anchored rehabilitation cohort data [5,13]. This review synthesises the available evidence into the stage-adapted cross-walk presented in Section 7, and three take-home messages follow.

First, rehabilitation should be stage-adapted. The integrated Lichtman radiographic, Schmitt MRI-perfusion, and Bain arthroscopic-cartilage information that now governs surgical reasoning [6,7] should likewise discipline rehabilitation timing, loading progressions, and patient counselling; longstanding observational series further indicate that a meaningful proportion of unoperated patients reach a symptomatic plateau despite radiographic progression [10,12], and this feature of the natural history belongs in the early-stage conversation.

Second, rehabilitation should be mechanism-anchored. The dart-throwing motion plane and dorsal-ligament-mediated sensorimotor reflexes deserve the same standing in rehabilitation reasoning that the integrated classification holds in surgical reasoning, with the caveat that direct Kienböck-cohort evidence for either remains lacking.

Third, rehabilitation should be measured. Even at the level of well-studied procedures such as radial shortening osteotomy and vascularised bone grafting, where meta-analyses now exist [81,82], pooled rehabilitation-relevant outcomes are constrained by inconsistent reporting; routine use of validated patient-reported (PRWE, QuickDASH) and performance-based (grip, three-plane motion, return-to-work) measures (with MCIDs honestly flagged as extrapolated from non-Kienböck cohorts) is the precondition for cumulative learning.

What this review adds is a synthesis matrix; what it does not provide is a prospectively validated algorithm. The translational priority is the publication of procedure-specific, JHT-style protocol papers and of CHT-led prospective cohorts in which the matrix presented here can be tested, refined, or refuted.

## Figures and Tables

**Table 8:** Stage cross-walk integrating osseous (Lichtman), articular (Bain and Durrant), Kienböck-specific precedent (Innes and Strauch), tissue phase-of-healing (Skirven), and analogue criterion-based guidance (APTA distal-radius clinical practice guideline 2024) for each reference-frame stage element.

Reference-Frame Stage Element	Lichtman et al. 2010/2017 [4,6] (Osseous)	Bain et al 2011 [43] (Articular)	Innes & Strauch SR	Skirven 7th Ed. Phase of Healing	APTA DR-CPG 2024 (Analogue)
Pre-collapse, lunate-overload symptoms	Stage I (BME, normal radiograph)	Vascular phase	Early-disease conservative track	Inflammatory → early proliferation	Phase 1: Protection
Early radiographic changes, no collapse	Stage II	Vascular to osseous transition	Continuation of early-disease track	Proliferation	Phase 1–2: Protection to Mobilisation
Joint-levelling/VBG decision point	Stage IIIA (no fixed deformity)	Osseous phase	Not addressed	Proliferation → remodelling	Phase 2: Mobilisation
Post-osteotomy phase	Stage IIIA stable	Osseous phase	Not addressed	Remodelling	Phase 2–3
Chondral compromise/Schmitt C perfusion	Stage IIIB	Chondral phase	Not addressed	Remodelling (load-axis-protected)	Phase 3: Strengthening
Salvage decision (PRC/4CF/TWA)	Stage IV	Chondral/pan-carpal	Not addressed	Remodelling	Phase 3–4
Return-to-function phase	Not applicable	Not applicable	Not addressed	Maintenance	Phase 4: Return to function

## Data Availability

No new data were created or analysed in this study.
